# Association of Physical Fitness and Diet with Body Weight in Austrian Adolescents

**DOI:** 10.3390/nu16234209

**Published:** 2024-12-05

**Authors:** Clemens Drenowatz, Gerson Ferrari, Tena Matolic, Maria do Carmo Greier, Klaus Greier

**Affiliations:** 1 Division of Sport, Physical Activity and Health, University of Education Upper Austria, 4020 Linz, Austria; 2 Escuela de Ciencias de la Actividad Física, el Deporte y la Salud, Universidad de Santiago de Chile (USACH), Santiago 9170022, Chile; gersonferrari08@yahoo.com.br; 3 Faculty of Health Sciences, Universidad Autónoma de Chile, Providencia 7500912, Chile; 4 Faculty of Kinesiology, University of Zagreb, 10000 Zagreb, Croatia; tena.matolic@kif.unizg.hr; 5 Division of Nutrition and Health, Private Educational College (KPH-ES), 6422 Stams, Austria; maria.greier@kph-es.at; 6 Department of Otorhinolaryngology, Head and Neck Surgery, Medical University Innsbruck, 6020 Innsbruck, Austria; 7 Department of Sports Science, Leopold-Franzens University Innsbruck, 6020 Innsbruck, Austria; nikolaus.geier@uibk.ac.at

**Keywords:** motor competence, physical activity, eating behavior, dietary pattern, body composition, youth

## Abstract

Background/Objectives: Physical fitness and diet along with body weight are key determinants of health. Excess body weight, poor dietary choices, and low physical fitness, however, are becoming increasingly prevalent in adolescents. In order to develop adequate intervention strategies, additional research on potential interaction effects of these entities is needed. Therefore, this study examined the combined association of physical fitness and diet with body weight in Austrian adolescents. Methods: A total of 164 (56% male) adolescents between 11 and 14 years of age completed the German Motor Test, which consists of eight items that assess cardiorespiratory endurance, muscular endurance and power, speed and agility, flexibility, and balance, along with body weight and height measurements. Additionally, participants completed a standardized food frequency questionnaire. Results: Spearman correlation analyses showed an inverse association between physical fitness and processed foods consumption (rho = −0.25, *p* < 0.01), while sweet consumption was positively associated with physical fitness (rho = 0.17, *p* = 0.03). No significant interaction effects between diet and physical fitness on body weight were observed. However, both higher physical fitness and greater sweet consumption were associated with lower body weight (*p* < 0.01). Conclusions: The present study emphasizes the independent and combined interactions of key correlates of health. It also suggests that high fitness may offset detrimental effects of poor dietary choices. In order to address potential health risks early in life and facilitate future health and well-being, it is important to monitor and control physical fitness, diet, and body weight during adolescence.

## 1. Introduction

Low levels of physical fitness and poor dietary habits are associated with various detrimental health outcomes, including excess body weight and non-communicable diseases (NCDs) [1,2]. While chronic health issues typically manifest in adulthood, their development often begins in childhood or adolescence [2,3]. The rising prevalence of obesity among children and adolescents is particularly concerning [4,5], as excess body weight in youth is difficult to reverse later in life [6]. Moreover, preventing childhood obesity is regarded as the most effective approach to reducing adult obesity and its related health conditions [7,8]. Individuals with excess body weight early in life face heightened risk for psychological disorders and social difficulties, as well as morbidity and mortality of various non-communicable diseases such as cardiovascular and metabolic diseases, respiratory problems, and some cancers [9,10]. Given the lack of success in tackling the obesity epidemic and its significant impact at the individual level as well as upon the health care system [11], it is considered one of the greatest public health challenges of the 21st century. Even though obesity is a multifactorial chronic disease [12], lifestyle choices play a key role in excess body weight [13,14]. Particularly, alterations in global food supply and a reduction of physical activity at work and during leisure time are central to the increasing prevalence of obesity [15,16,17].

Physical fitness is another important indicator of both health and health-related quality of life [18,19], as low physical fitness during adolescence has been associated with various chronic disease risk factors that can persist into adulthood [20,21]. In fact, cardiorespiratory fitness has been shown to be a more powerful predictor of mortality risk than many other common health risk factors [22,23,24]. Nevertheless, physical fitness has been declining over the last several decades [25,26], and ensuring adequate physical fitness during childhood and adolescence is crucial for the prevention of chronic diseases and maintenance of overall health [19]. Given its association with physical activity [24,27], physical fitness is further considered an important indicator for adolescents’ lifestyles. Similarly, dietary habits have been associated with improved quality of life, cognitive functions, and mental health as well as reduced risk of various diseases [28,29,30,31]. Despite these well-documented benefits, available data indicates poor diet in children and adolescents along with low levels of physical activity and physical fitness [32,33,34].

Adolescence presents a critical window of opportunity to influence long-term health and well-being. This period is marked by significant psychological and physiological changes, and it is well-established that many health behaviors formed during childhood and adolescence persist into adulthood [35,36]. Dietary habits during childhood and adolescence, for example, are significant predictors of dietary patterns in adulthood [37,38]. Similarly, physical fitness tends to track from young ages into later years [39]. Therefore, adopting healthy habits during childhood and adolescence can help with the prevention of excess body weight and associated comorbidities later in life [40,41,42]. In order to develop adequate health promotion strategies in this pivotal time of life, it is necessary to enhance the understanding of the interaction between key correlates of future health. Therefore, the aim of this paper is to examine associations of physical fitness, dietary patterns, and body weight in adolescents. This knowledge can help in determining whether health-related behaviors can be addressed individually or should be addressed in a combined effort.

## 2. Materials and Methods

The present study uses a cross-sectional design. Nine classes from grades six to eight were selected from Austrian middle schools, resulting in 172 eligible participants aged 11 to 14 years, all without cognitive, physical, or motor limitations. Parents received information about the nature of the study and provided written informed consent; participants provided oral assent at the time of data collection. The study protocol was approved by the Institutional Review Board of the University of Innsbruck (approval number: 31/2022) and the school board of the Federal State of Tyrol, Austria. All study procedures followed the ethical standards of the 2008 Declaration of Helsinki.

Body weight (kg) and height (cm) were measured during a physical education class by trained technicians according to standard procedures, with participants being barefoot and wearing gym clothes. Body weight was measured to the nearest 0.1 kg with a gauged body scale (SECA^®^ 803, Seca, Hamburg, Germany) and height was measured to the nearest 0.1 cm with a mobile stadiometer (SECA^®^ 217, Seca, Hamburg, Germany). Subsequently, body mass index (BMI, kg/m^2^) was calculated and converted to BMI percentiles (BMIPCT) based on the German reference system [43]. Participants with a BMIPCT above the 90th percentile were considered overweight/obese and participants with a BMIPCT below the 10th percentile were considered underweight.

Following anthropometric measurements, participants completed the German motor test (DMT6-18), which has been validated for children and adolescents between 6 and 18 years of age [44]. The DMT6-18 consists of eight test items that measure cardiorespiratory endurance, muscular endurance, muscular power, speed, agility, balance, and flexibility, which are the main components of health-related fitness [45]. Specifically, participants performed a 6-min run, push ups, sit ups, a standing long jump, a 20-m sprint, sideways jumping, backwards balancing and a stand-and-reach test during a single test session. All tests were administered following the guidelines provided in the test manual. The 20-m sprint was completed at the beginning and the 6-min run was completed at the end of the test session. The remaining six tests were completed in random order with sufficient break time between tests in order to avoid undue fatigue. Raw performance measurements were converted into age- and sex-normalized scores (*Z*-scores), with values between 97 and 103 indicating average performance for the corresponding age and sex [44]. The average of all *Z*-scores was used as an indicator for total fitness. Subsequently, sample-specific tertiles for total fitness were established for further analyses (low fitness: *Z* ≤ 100, moderate fitness: 100 < *Z* < 108, high fitness: *Z* ≥ 108)

Dietary information was obtained via a standardized food frequency questionnaire that has been used previously in Austrian adolescents [46]. The questionnaire was administered during regular class time by trained personnel. The participants were asked to report the frequency (days/week) of consumption of 42 foods, which were subsequently grouped into 17 food categories. These food categories were used in a principal component analysis, which identified six dietary factors with Eigenvalues > 1, explaining 64.0% of the total variance of dietary intake. Based on individual factor scores, participants were subsequently categorized as having a high or low score using a median split.

The factors were characterized by high loadings of the following food categories:

- Factor 1 (processed foods): frozen and fast food, energy drink, alcohol (Eigenvalue = 4.12; explains 24.23% of variance);
- Factor 2 (plant-based diet): fruits, nuts, soy milk (Eigenvalue = 2.01; explains 11.84% of variance);
- Factor 3 (healthy mixed diet): rice, bread/noodles, fish, vegetables, water (Eigenvalue = 1.49; explains 8.75% of variance);
- Factor 4 (animal-based diet): meat, eggs, milk (Eigenvalue = 1.14; explains 6.72% of variance);
- Factor 5 (sweets): sweets, jam (Eigenvalue = 1.08; explains 6.37% of variance);
- Factor 6 (supplements): dietary supplements (Eigenvalue = 1.04; explains 6.11% of variance).

Statistical analysis. Normal distribution was assessed using *Z*-scores for kurtosis and skewness between ± 3.29 to determine normality [47]. Descriptive statistics are presented as means with standard deviations for continuous variables and as prevalence rates for categorical variables. Chi-Square tests were performed to assess associations between categorical variables (i.e., sex distribution, across dietary categories, weight categories, and total fitness tertiles). Due to the lack of normal distribution for some physical fitness variables, Spearman correlation analyses were used to examine the association between dietary factor scores and physical fitness. The association of dietary factors and physical fitness tertiles with BMIPCT was examined using a 2 (diet factor) × 3 (physical fitness) ANOVA since BMIPCT followed a normal distribution. Additionally, logistic regression was performed to assess the association of dietary factors and physical fitness with the likelihood of being overweight/obese. All statistical analyses were conducted for the total sample and separately for boys and girls using SPSS 27.0 (IBM, Armonk, NY, USA), with the statistical significance set at *p* < 0.05.

## 3. Results

A total of 164 adolescents (55.5% male) with an average age of 12.9 ± 1.2 years provided complete data. The prevalence of overweight/obese adolescents was 18.9% (*n* = 31), while the prevalence of underweight adolescents was 7.9% (*n* = 13). Compared to girls, boys were significantly older (*p* < 0.01), which led to significant sex differences in body weight and height (Table 1). However, no significant sex differences were found in BMIPCT and the prevalence of overweight/obese adolescents (16.4% in girls and 20.9% in boys).

The mean *Z*-score for total fitness across the entire sample indicated above-average fitness levels. Specifically, mean scores for push-ups, long jump, sideways jumping, 20-m sprint, and balance were above average, while mean scores for the 6-min run and sit ups were below average. Stand-and-reach performance was average based on the sample mean. Girls outperformed boys in sit ups and the stand-and-reach test, while boys achieved better results in the 20-m sprint (Table 1). However, there was no significant difference in total physical fitness between boys and girls, and sex distribution did not differ across total fitness tertiles (chi square = 3.25; *p* = 0.21).

### 3.1. Diet Factors and Physical Fitness

Girls displayed a significantly higher factor score for a plant-based diet compared to boys (0.19 ± 1.1 vs. −0.16 ± 0.9; *p* = 0.03), while their factor scores for processed foods and animal-based diet were lower (−0.26 ± 0.88 vs. 0.21 ± 1.0 and −0.26 ± 1.1 vs. 0.21 ± 0.9; *p* < 0.01, respectively). No sex differences were observed for the factor scores for healthy mixed diet, sweets, and supplements. In line with these results, the prevalence of boys was significantly higher in the high processed food group and the high animal-based diet group (chi square = 4.17; *p* = 0.04 for both), while no sex differences were observed in the other dietary factor groups (Table 2).

Correlation analyses between dietary factors and physical fitness in the whole sample indicated a significant inverse association of total physical fitness with processed foods (Spearman’s rho = −0.25, *p* < 0.01), and a significant positive association with the factor sweets (Spearman’s rho = 0.17, *p* = 0.03). More specifically, processed foods consumption was inversely associated with the 6-min run, push-ups, sit-ups, sideways jumping and balancing while sweets consumption was positively associated with sprint performance (Table 3). In addition, a higher factor score for plant-based diet was associated with better 6-min run performance, while a higher factor score for animal-based diet was associated with lower sit-ups performance. No significant associations were found between physical fitness and factor scores for a healthy mixed diet, or supplements.

Sex-specific correlation analyses revealed significant inverse associations of total physical fitness with processed foods in boys (Spearman’s rho = −0.26, *p* = 0.01). Specifically, performance at the 6-min run (Spearman’s rho = −0.27, *p* < 0.01), push-ups (Spearman’s rho = −0.30, *p* < 0.01) and sideways jumping (Spearman’ rho = −0.34, *p* < 0.01) were inversely associated with processed foods. Sweet consumption, on the other hand, was positively associated with total physical fitness (Spearman’s rho = 0.33, *p* < 0.01) among boys. Specifically, there was a positive association with the 6-min run (Spearman’s rho = 0.30, *p* < 0.01), push-ups (Spearman’s rho = 0.23, *p* < 0.05), sit-ups (Spearman’s rho = 0.23, *p* < 0.05), sprint (Spearman’s rho = 0.30, *p* < 0.01), and balance (Spearman’s rho = 0.22, *p* < 0.05). No other significant associations between dietary factors and physical fitness were observed in boys. Among girls, no significant associations between dietary factors and total physical fitness were observed. Nevertheless, higher factor scores for processed foods were significantly associated with lower balance performance (Spearman’s rho = −0.25, *p* = 0.03).

### 3.2. Association of Dietary Factors and Physical Fitness with Body Weight

**Figure 1:**
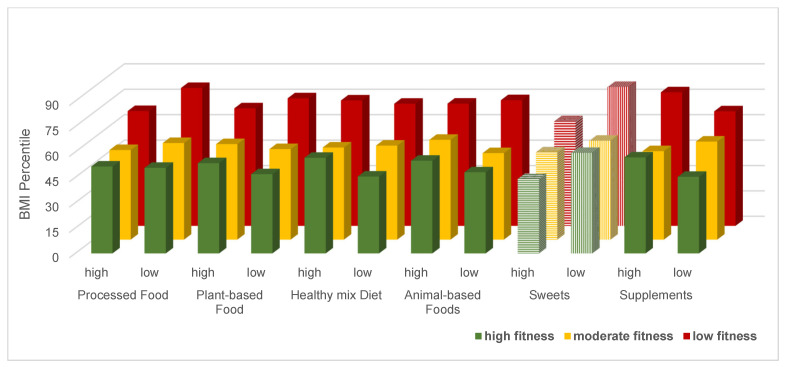
Combined association of diet and physical fitness with BMI percentile. Values are mean. Dashed color indicates significant main effect for sweets consumption on BMI percentile.

There were no significant interaction effects observed between diet and physical fitness on BMIPCT for the whole sample (Figure 1). There was, however, a significant main effect for physical fitness, with participants displaying high and moderate physical fitness levels having significantly lower BMIPCT than their peers with low physical fitness (*p* < 0.01). Associations between dietary factors and BMIPCT were limited, except for a higher sweet consumption being associated with a lower BMIPCT (Table 4).

**Figure 2:**
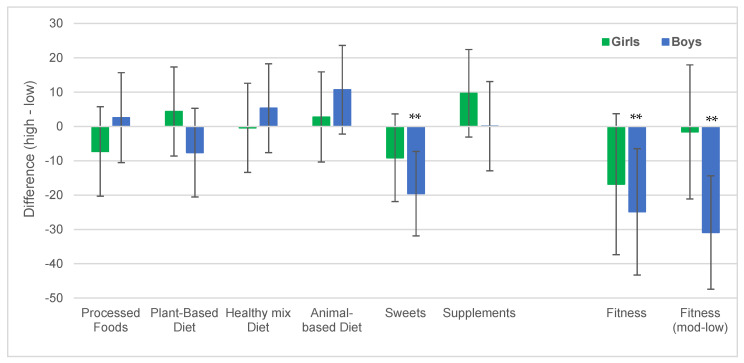
Differences in BMIPCT between high and low dietary factor scores and high and low and as well as moderate and low fitness scores among girls and boy. Values are mean differences with 95% CI. ** significant difference at *p* < 0.01.

Sex-specific analyses showed similar results in boys. Higher physical fitness and higher sweet consumption was associated with lower BMIPCT. In girls, on the other hand, no significant associations of physical fitness or diet with BMIPCT were observed (Figure 2).

The results of logistic regression further showed an increased risk for overweight/obesity with lower physical fitness, particularly in boys (Table 5). In addition, higher sweet consumption was associated with a lower risk for overweight/obesity in boys. In girls, however, no significant associations between dietary factors and weight status were observed.

## 4. Discussion

The present study examined the association of diet, physical fitness, and body weight in Austrian adolescents and found several significant results. Among the entire sample population, (i) cardiorespiratory endurance and strength were inversely associated with diets high in processed foods and animal products, (ii) a plant-based diet was associated with higher cardiorespiratory endurance, (iii) higher physical fitness was associated with lower body weight, and (iv) high sweet consumption was associated with lower body weight. Despite significant main effects of diet and physical fitness on body weight, there were no significant interaction effects. Significant results were evident particularly among boys, where total physical fitness, cardiorespiratory fitness, muscular strength, and agility were found to be inversely related with a diet high in processed foods, while these components of physical fitness were positively associated with sweet consumption along with speed and balance.

As in this study, potential beneficial influences of specific dietary patterns on physical fitness have been reported in previous studies. For example, adherence to the Mediterranean diet, which is based on plant-based foods and limited intake of processed foods, has been associated with higher physical fitness—particularly cardiorespiratory fitness—in children and adolescents [48,49,50,51], with stronger effects observed in boys compared to girls [52]. A review on adherence to the Mediterranean diet in children and adolescents further showed a direct association with physical activity [53], which is a key correlate of physical fitness [54,55]. However, our study also showed a positive association between sweet consumption and physical fitness, which is in contrast to the previous studies [56,57]. A possible explanation may be that higher fitness is associated with increased PA, particularly of higher intensities [58,59], which could offset the potential detrimental effects of high sweet consumption due to a higher demand for carbohydrates. This aspect was shown by a study where preference for sweet foods increased after physical exercise [60]. Similarly, sports participation has been associated with a higher consumption of sports drinks [61,62], which usually contain a considerable amount of sugar.

The direct association between sweet consumption and physical fitness observed in this study may also help explain the inverse relationship between body weight and sweet consumption. Previous research by Lahoz-Garica et al. also showed that cardiorespiratory fitness mediates potential negative effects of diet on body fat [63], which may have contributed to the lack of an association between other dietary factors and body weight in this study. Even though high sugar intake is generally associated with increased body weight, such research focused predominantly on sugar-sweetened beverages [64,65], whereas consumption of sweet bakery has been associated with a lower risk of overweight/obesity [65]. Other studies also reported no association between dietary patterns and body weight in adolescents [48,49], and a study by Wilkie et al. even found an increased risk for overweight or obesity with a high healthy diet score [66]. A recent review, therefore, concluded that the ideal macronutrient composition for weight management remains unclear, and that total caloric intake in relation to energy expenditure has a greater impact on body weight rather than a specific diet composition [67]. Accordingly, engaging in physical activity and limiting the use of electronic devices have been recommended as the most potent modifiable behaviors for weight management in adolescents [68]. It should also be considered that adolescents engaging in regular physical activity may be more likely to consume snacks due to a lack of time for full meals [69]. However, snacking behavior, which potentially contributes to higher sweet consumption, has not been associated with higher body weight either, and may even reduce the risk for obesity [70].

Consistent with our findings, low physical fitness has been regularly associated with higher body weight [71], and high fat mass in particular has been associated with lower physical fitness [52]. This is particularly important because maintaining adequate physical fitness at young ages is considered essential for long-term health and well-being [19], especially as low physical fitness levels tend to track into adulthood [39]. High physical fitness during adolescence has also been associated with better self-rated health [72,73], and it facilitates engagement in physical activity later in life [74,75]. Similarly, higher physical fitness, as observed in the present study, may be attributed to increased levels of physical activity [76,77], underscoring the importance of physical fitness for adolescent health. However, it should be considered that many fitness tests require individuals to move their own body weight, which puts heavier subjects at a disadvantage and may contribute to lower fitness scores. On the other hand, daily activities also require movement of one’s body weight, so fitness scores adjusted for body weight may not provide adequate information on individual’s functional capacity. Nevertheless, it is evident that physical fitness is an important contributor to current and future health [45]. Higher physical fitness has been associated with better self-rated health in adolescents [72,73], and cardiorespiratory fitness in particular has a stronger association with mortality risk than other well-established risk factors, including lipid abnormalities, smoking, and hypertension [23,24,78].

There are some limitations of the present study that should, however, be considered when interpreting the findings. Given the cross-sectional nature of the study, causal relationships cannot be determined. The homogenous sample and limited number of participants may also limit generalizability of these findings, particularly as the majority of the participants displayed average-to-above-average fitness levels. Furthermore, dietary information was obtained via self-report, which holds an inherent risk for reporting bias. The questionnaire also focused on the frequency of consumed foods rather than the amount. Accordingly, there was no information on caloric intake, which is critical in weight management. Physical fitness, however, was assessed with a validated and widely used test battery, and body weight and height were measured according to standard procedures. Additional research, nevertheless, is needed to enhance the understanding of the role of physical fitness and diet in weight management. In particular, research on potential interaction effects in adolescents with low physical fitness is warranted. Furthermore, a larger sample size and more detailed information on dietary habits in addition to food consumption may allow for a more sophisticated analytic approach. Given the complexity, recent reviews also emphasized the need to further explore the clustering of heath behaviors, such as physical activity, sleep and diet [79,80].

## 5. Conclusions

This research underscores the critical role of physical fitness in weight management during childhood and adolescence, suggesting it may even mitigate the effects of poor dietary choices. The associations between specific dietary patterns, physical fitness, and body weight further emphasize the interconnectedness of these important health correlates. Given that many adolescents are displaying poor health behaviors, there is a need to continue the evaluation and monitoring of body composition, physical fitness, and dietary habits at young ages in order to develop adequate preventive measures to ensure future health and well-being.

## Figures and Tables

**Table 1:** Anthropometric characteristics and physical fitness for the total sample and separately for girls and boys. Values are means with standard deviation.

	Total Sample (N = 164)	Girls (N = 73)	Boys (N = 91)
Age (years) **	12.9 ± 1.2	12.7 ± 1.1	13.1 ± 1.2
Height (cm) **	161.3 ± 8.9	158.7 ± 6.7	163.4 ± 9.9
Body weight (kg) *	53.8 ± 14.4	51.3 ± 10.0	55.9 ± 16.9
BMI Percentile	59.6 ± 29.4	61.3 ± 27.5	58.2 ± 31.0
6 Min Run (Z)	94.1 ± 11.2	99.1 ± 8.6	90.2 ± 11.5
Push Ups (Z)	108.1 + 11.2	109.9 + 10.0	106.6 ± 11.9
Sit Ups (Z) *	95.2 ± 8.7	97.2 ± 6.9	93.6 ± 9.7
Longjump (Z)	103.3 ± 11.7	103.6 ± 10.6	102.9 ± 12.5
Side Jumps (Z)	113.7 ± 10.9	114.3 ± 10.9	113.2 ± 10.9
20-m Sprint (Z) *	105.8 ± 12.4	103.3 ± 12.5	107.8 ± 12.1
Balance (Z)	105.2 ± 10.0	105.4 ± 8.4	105.0 ± 11.2
Stand and Reach (Z) *	99.9 ± 11.5	102.5 ± 10.8	97.9 ± 11.7
Total Fitness (Z)	103.3 ± 7.7	104.6 ± 7.0	102.3 ± 8.1

* significant sex difference at *p* < 0.05; ** significant sex difference at *p* < 0.01.

**Table 2:** Prevalence of boys and girls in high and low dietary factor groups.

		Processed Foods	Plant-Based Diet	Healthy Mixed Diet	Animal-Based Diet	Sweets	Supplements
Boys	High	57.1%	46.2%	47.3%	57.1%	50.5%	51.6%
	Low	42.9%	53.8%	52.7%	42.9%	49.5%	48.4%
Girls	High	41.1%	53.4%	53.4%	41.1%	49.3%	47.9%
	Low	58.9%	46.6%	46.6%	58.9%	50.7%	48.4%

**Table 3:** Association between dietary factors and components of physical fitness (expressed in *Z*-scores). Values are Spearman’s rho.

	6 Min Run	Push Ups	Sit Ups	Long Jump	Side Jumps	Sprint	Balance	Flexibility
Processed Foods	−0.33 **	−0.24 **	−0.17 *	−0.11	−0.25 *	−0.03	−0.16 *	−0.04
Animal-based diet	−0.14	−0.12	−0.17 *	−0.02	−0.06	−0.12	0.04	−0.07
Plant-based diet	0.18 *	0.02	0.04	0.05	0.05	0.04	0.05	0.05
Sweets	0.12	0.11	0.10	0.12	0.08	0.19 **	0.15	0.06

* significant correlation at *p* < 0.05; ** significant correlation at *p* ≤ 0.01.

**Table 4:** BMIPCT by dietary factor and physical fitness group. Values are means ± SD.

	High	Moderate	Low	*p*-Value
Processed Foods	58.5 ± 29.7	N/A	60.7 ± 29.3	0.63
Plant-based Diet	58.6 ± 28.8	N/A	60.6 ± 30.2	0.66
Healthy mixed Diet	61.1 ± 30.3	N/A	58.1 ± 28.7	0.52
Animal-based Diet	62.8 ± 27.9	N/A	56.3 ± 30.7	0.16
Sweets	52.1 ± 30.7	N/A	67.1 ± 26.2	<0.01
Supplements	61.7 ± 30.6	N/A	57.5 ± 28.3	0.36
Physical Fitness	50.9 ± 25.5	54.7 ± 28.7	73.1 ± 29.3	<0.01

N/A—not applicable.

**Table 5:** Association of physical fitness and dietary factors with overweight/obesity in the total sample and separately for girls and boys. Values are β-coefficients with 95% confidence intervals.

	Physical Fitness	Processed Foods	Plant-Based Diet	Healthy Mixed Diet	Animal-Based Diet	Sweets	Supplements
Total Sample	0.85 ** (0.79; 0.91)	0.83 (0.52; 1.32)	0.65 0.39; 1.089	1.32 (0.87; 2.02)	1.43 (0.91; 2.25)	0.71 (0.46; 1.09)	1.22 (0.84; 1.77)
Girls	0.90 (0.81; 1.01)	0.45 (0.12, 1.75)	0.63 (0.27; 1.50)	1.81 (0.82; 3.97)	1.11 (0.50; 2.48)	1.19 (0.56; 2.52)	1.48 (0.90; 2.44)
Boys	0.82 ** (0.74; 0.91)	0.88 (0.52; 1.51)	0.81 (0.38; 1.74)	1.21 (0.69; 2.11)	1.40 (0.74; 2.64)	0.56 * (0.32; 0.98)	1.02 (0.52; 1.97)

* significant association with overweight/obesity at *p* < 0.01; ** significant association with overweight/obesity at *p* < 0.01.

## Data Availability

The data presented in this study are available on request from the corresponding author privacy reasons.
